# Muscle Ultrasonographic Elastography in Children: Review of the Current Knowledge and Application

**DOI:** 10.3390/children8111042

**Published:** 2021-11-12

**Authors:** Agnieszka Cebula, Maciej Cebula, Ilona Kopyta

**Affiliations:** 1 Department of Paediatric Neurology, Faculty of Medical Sciences in Katowice, Medical University of Silesia in Katowice, Medykow Str 16, 40-752 Katowice, Poland; ikopyta@sum.edu.pl; 2 Department of Radiodiagnostics, Invasive Radiology and Nuclear Medicine, Department of Radiology and Nuclear Medicine, Faculty of Medicine in Katowice, Medical University of Silesia in Katowice, Medykow Str 14, 40-752 Katowice, Poland; mcebula@sum.edu.pl

**Keywords:** ultrasonographic elastography, neuromuscular disease, muscle, children

## Abstract

Ultrasonographic elastography is a relatively new imaging modality for the qualitative and quantitative assessments of tissue elasticity. While it has steadily gained use in adult clinical practice, including for liver diseases, breast cancer, thyroid pathologies, and muscle and tendon diseases, data on its paediatric application is still limited. Moreover, diagnosis of muscular diseases in children remains challenging. The gold standard methods, namely biopsy, electroneurography, and electromyography, are often limited owing to their invasive characteristics, possible contraindications, complications, and need for good cooperation, that is, a patient’s ability to perform certain tasks during the examination while withstanding discomfort, which is a significant problem especially in younger or uncooperative children. Genetic testing, which has broad diagnostic possibilities, often entails a high cost, which limits its application. Thus, a non-invasive, objective, repeatable, and accessible tool is needed to aid in both the diagnosis and monitoring of muscle pathologies. We believe that elastography may prove to be such a method. The aim of this review was to present the current knowledge on the use of muscle elastography in the paediatric population and information on the limitations of elastography in relation to examination protocols and factors for consideration in everyday practice and future studies.

## 1. Introduction

The diagnosis and monitoring of neuromuscular diseases remain a challenge despite the emerging role of genetic testing in this field. Vital limitations in diagnosis and monitoring are particularly relevant in the paediatric group owing to the high costs of genetic tests and invasiveness of gold standard tests (electromyography (EMG), electroneurography (ENG), and biopsy). Both ENG and EMG require good patient compliance (ability to simultaneously withstand discomfort and relax or contract muscles on demand), may lead to complications like most invasive procedures, and are often limited by the need to ensure patient safety and health-related contraindications. Thus, a new, non-invasive, affordable, and objective test is urgently needed. We believe that elastography may prove to be such a tool in the future, as it has already been applied in hepatological, endocrinal, and oncological diagnostics [1,2]. Although far more studies on muscle elastography have been conducted in the adult population than in children, results from the former cannot be simply applied to the latter. Thus, the aim of this study was to present existing elastography modalities, their limitations, and applications in paediatric muscle-related disorders.

### Technical Aspects of Elastography

Elastography is an assessment method based on the elastic properties of soft tissues. A few modalities based on magnetic resonance (MR) and ultrasonography (US) already exist. However, we limited the present study to US modalities, as they are easier to access in everyday clinical work, provide real-time metrics for most cases, and are less costly. Studies that compared elastography from MR and different US methods in children are scarce, and their results remain inconsistent [3,4,5].

**Figure 1:**
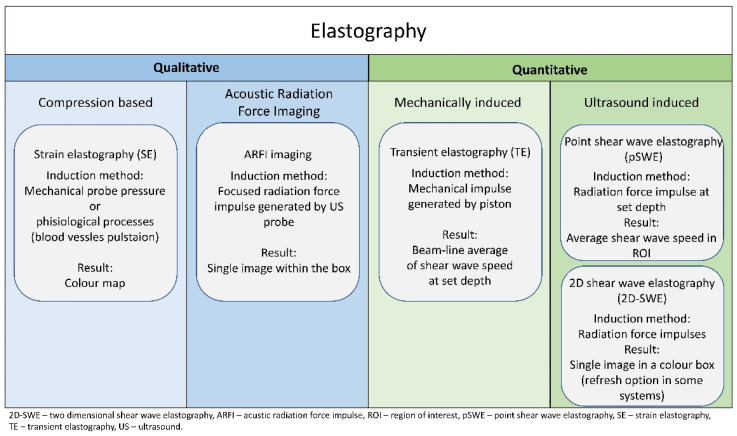
Simplified division of elastographic methods.

The main US elastographic modalities are strain elastography (SE), acoustic radiation force impulse (ARFI), transient elastography (TE), and shear wave elastography (SWE). In general, the main distinctive differences are the method of stress application, detection of tissue deformation, and characteristics of gained data (qualitative vs. quantitative). A simplified classification of elastographic methods is presented on Figure 1. One of the most important impediments in elastographic research analysis is provider-dependant varieties, subdivision of modalities, and inconsistency in nomenclature.

SE is currently widely available, as it is offered by most leading manufacturers of US devices. The qualitative measurement is based on the amount of strain that is mechanically induced by either the mechanical pressure of the US probe or physiological processes such as blood vessel pulsation. The effect is presented as a colour map, with some vendors offering software for semi-quantitative analysis such as strain ratio (SR) calculation. Among the SE methods, a Hitachi-patented real-time elastography device has gained the most interest. It implements the extended combined autocorrelation method, an algorithm that correlates in both axial and lateral directions and produces an elasticity image in real time. Despite the implementation of various quality-control systems, the method is still heavily operator-dependant [1,6].

ARFI imaging is a Siemens-patented qualitative method based on the focused radiation force impulse produced by the US probe, whose displacement is evaluated at the set depth. The results are shown as a single image within the box. The attainability with systems by a single manufacturer is a valid availability limitation [1].

TE is the first quantitative method designed by Echosens for the evaluation of liver fibrosis and steatosis. A specifically designed piston induces a mechanical impulse, and US is used for beam-line average measurement of the resulting shear wave speed. Owing to its design, this method has not played a major role in muscular evaluation [1].

SWE is a group of methods that can be roughly divided into point and two-dimensional (2-D) SWEs, with further subdivision according to the method of force application. From a practical point of view, all these methods measure shear wave speed and offer quantitative results expressed in meters per second, which are subsequently converted to kilopascals by using the Young modulus. One of the main limitations is the assumption of homogeneity of the wave propagation medium, which is usually not the case with muscles. Other limitations include the manufacturer-dependent method of induction and calculation of shear wave speed, which make a direct comparison of vendor results impossible [1,7,8]. SWE and ARFI may also be affected by the depth of examined tissues [7,9,10,11]. As in the paediatric population, tight muscle thickness was reported to be up to 45 mm. Thus, SWE might have a broader application in this age group [12]. In general the most commonly used devices are Aixplorer (Supersonic Imagine, Aix-en-Provence, France), which supports 2-D SWE, and Acuson (S2000/S3000; Siemens, Washington, DC, USA), which generates ARFI [13].

## 2. Materials and Methods

**Figure 2:**
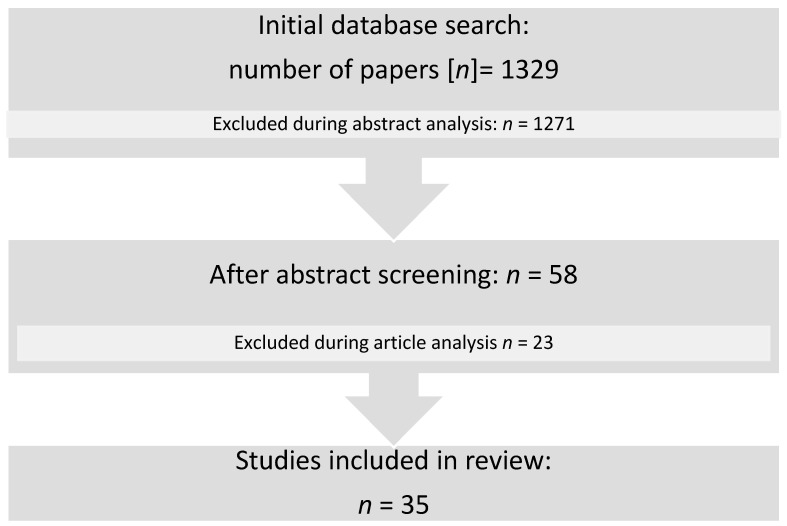
Flow diagram of the literature search.

A thorough literature search on the PubMed database was performed using the MeSH terms “elasticity imaging techniques/methods”, “child”, and “muscle”, and search words with the search operator AND (“elastography”, “child”, “muscle”, and “children”). After the initial search of manuscripts from 2012 to 2020, 1329 studies were found. We limited the number of studies to 58 by manually checking articles and their abstracts. In addition, their bibliographies were analysed and checked. Studies that did not involve children, those that analysed only different muscular tissues (including studies on tendons only), those that had only the abstract available, duplicate articles, short reviews of the general use of elastography, those that involved only MR elastography, and those that were in languages other either Polish or English were excluded. Finally, 35 articles were included in this study. A summary of the process is presented in Figure 2.

The results presented were divided into two parts as follows: one is the technical and demographic aspects that influence the results, and the other is current knowledge on muscle elastography results in different clinical problems in children.

## 3. Factors Influencing Elastography

The following subparagraphs focus on factors affecting the elastography results that are not connected to the patient’s disease. The sex, age, anthropometry, muscle stretching, tissue compression and operator-related reliability are discussed in turns.

### 3.1. Differences Related to Patient Sex

While multiple factors may influence muscular status, data regarding the association between patient sex and elastography results are too limited to form a clear conclusion. However, few studies have observed no sex-related differences between modalities in small children.

Brandenburg et al. found no significant sex-related difference in a study of SWE of the gastrocnemius muscle (GCM) in 20 healthy children aged 2 to 12 years. All the patients were in their prepubertal age [14]. No significant difference in ARFI was found in a study in 12 children with cerebral palsy (CP) aged 6 to 14 years and a study of SWE of the GCM in 86 patients from different age groups [15,16]. The latter involved 27 children (6–12 years) and 59 adults, but the authors did not assess the association of sex to muscles stiffness separately for the different age groups [15].

On the other hand, Koppenhaver et al. evaluated 130 adult patients to study factors that affect lumbar muscle stiffness on SWE. In this study, significant differences were found between men and women in all the muscles evaluated, with larger shear modules in the men. The authors estimated that the values for the male group were approximately 20% higher than those for the female group across all the examined lower back muscles in both the relaxed and contracted states [16]. Significant sex-related differences in ARFI and SWE findings were also described in other studies in adult populations [9,17,18]. One study of 42 healthy children found that there was significant difference between male and female’s rectus femoris muscle but only at rest [19].

This sex-related disparity in elastography results between age groups and studies has raised the question of whether the observed changes might be dependent on maturity and the location and type of the examined muscles. More data are needed to clarify this.

### 3.2. Age-Related Differences

Increase in muscle stiffness with age was clearly described in some adult studies [9,17]. A similar tendency was observed in paediatric studies, with some minor deviations [14,15,20,21,22].

Liu et al. evaluated the GCMs of 86 healthy volunteers, divided into three age groups (paediatric: <16 years, middle-aged: 30–40 years, and old: >55 years). While no statistically significant differences were found in the plantar flexion (PF) and neutral positions of the feet, a significant increase in muscle stiffness in dorsiflexion (at all angles of the ankle from 10° to 30°) was observed with age [15]. In a study by Wenz et al. that compared the SE results of the upper and lower limb muscles in young adults (22 patients, aged 20–30 years) and children (21 patients, aged 2–12 years), significant differences were found between the two groups [20]. Brandenburg et al. assessed muscle changes in 20 children (aged 2–12 years) and found that muscle stiffness increased with age but it did not reach statistical significance [14].

An interesting trend was described in a study of Achilles tendons in Turkish paediatric populations with and without CP. A significant difference in age was found, but while the SR increased with age in the CP group (*p* < 0.001), it showed an inverse correlation with age in the healthy group (*p* = 0.038). The authors concluded that changes in muscle stiffness not only occur due to ageing but also may show specific patterns of ageing in different diseases [22]. In a study in patients with Duchenne muscular dystrophy (DMD) aged 5 to 24 years, results were incoherent in that while a statistical correlation was found between age and GCM stiffness in the DMD group, no correlation was found in the healthy group or for other muscles in the DMD group [21].

### 3.3. Differences Related to Anthropometry and Anisotropy

No relationship was found between muscle stiffness and calf circumference in healthy subjects. A tendency for decreasing muscle stiffness in relation to increasing BMI was observed but did not reach statistical significance. Range of movement and foot dominance did not influence muscle stiffness [14]. Another study on children population presented incoherent results: while higher BMI was related with decrease of biceps brachii long elasticity immediately after exercise, but none at rest, for rectus femoris muscle it was the other way around. The authors found significant rise of elasticity with higher BMI but only at rest and not following exercise [19]. In the adult population in the study of Koppenhaver et al., a clear association was found between BMI, self-assessed activity level, and SWE results [16].

Gennison et al. studied the effect of a muscle’s anisotropy, i.e., changes in mechanical properties along with the direction of measurement [23]. The longitudinal direction is recognized as the most relevant, as the muscle mechanical properties change with its lengthening and shortening [21,24]. The impact of the muscle anisotropy is still a subject of intensive research, with almost a hundred papers published annually. Yet, probably due to mentioned study results–authors of cited papers used longitudinal direction in their studies.

### 3.4. Passive Muscle Stretching Influences Study Results

Few studies focused on muscle stretching-related changes in muscle elasticity. Most of these studies assessed GCM changes as the effects of ankle joint dorsiflexion (DF), PF, and neutral position. Even though clear methodological differences (regarding the exact ankle angle measured, knee flexion, the study protocol used, and the tools used to ensure planned feet position) were present, the results were coherent; passive stiffness increased as a result of increased DF angle [14,15,25]. In a study of SWE of the anterior tibialis (AT) muscle and GCM in hemiplegic patients, Lee et al. confirmed that ankle angle had significant effects on both muscles. The study demonstrated a quadratic relationship between ankle angle and SW speed [26]. In a study by Brandenburg et al., muscle stiffness on SWE at 10° dorsiflexion was 4 times higher than at 20° PF [14]. Lacourpaille et al. proved that while muscle stiffness differed between patients with DMD and their healthy peers in 5 of 6 examined muscles during muscle stretching, this difference was present in only 3 muscles when no stretching was applied [21]. Thus, we may conclude that muscle stretching substantially changes muscle stiffness, especially in neuromuscular diseases, and not taking it into consideration may lead to errors in the study results and diagnosis.

Caliskan et al. focused on different aspects of muscle stretching. They studied whether the duration of passive muscle stretching affected muscle elasticity on SWE. Twenty male athletes aged 12 to 16 years were recruited. SWE was performed before and after 2 min (group 1) and 5 min (group 2) of passive stretching. While in the first group, no significant differences in pre-stretching results were found, significant reduction in muscle stiffness was observed in group 2 after 5 min. Even though the study population was small and limited to one sex, apart from the practical conclusions for sport medicine and physiotherapy, this study showed that the elastography examination protocol should carefully consider passive muscle stretching [25].

### 3.5. Exercise and Effort’s Effect on Muscle Elasticity

Apart from mentioned above effect of stretching, effort and exercise lead to changes seen in elastography on their own. In a study of 40 paediatric patients, Berko et al. proved that results of strain elastography before and immediately after leading to fatigue exercise significantly differed. The post-exercise elasticity of both biceps brachii long and rectus femoris muscle was lower [19]. This effect must be taken into account while planning clinical use of muscle elastography.

### 3.6. Influence of Tissue Compression on Elastography

As muscle tissue reacts to compression and passive elongation, with possible exacerbation of the reaction in some neuromuscular diseases, the question of whether force applied by the US probe changes the elastography results remains valid. In most of the studies included in this analysis, researchers decided to minimise the effect by either performing the study with as little compression as possible or reducing the distortion of the subcutaneous soft tissue with the use of a generous amount of US gel [4,14,18,27,28]. The latter solution might have an important limitation as seen in a study in 23 adult patients by Alfuraih et al. The authors compared SWEs of the vastus lateralis muscle with minimal pressure applied to the skin and with “standoff gel” applied at a minimum thickness of 5 mm. While no significant changes in mean shear wave speed was found between the groups, the reliability quantified by intraclass correlation coefficient (ICC) decreased from near-perfect agreement (ICC = 0.83) to the margin of substantial agreement (ICC = 0.62) in the gel method [7]. Further studies on the compression effect of the probe on elastography results are needed.

### 3.7. Operator-Related Reliability

A few studies assessed inter- and intra-operator reliability as moderate to excellent regardless of the muscle evaluated and modalities used (ICC, from >0.6 to >0.9) [24,29,30,31] A. While no relationship between BMI and elastography results in children were described by Linek et al., they found that the thickness of fat tissue above the lateral abdominal muscles influenced the reliability of the results of their study. Thinner fat layers (<5 mm on average) had positive influences on inter-rater reliability. In addition, the side of the examined muscle carried consequences as well. Worse inter- and intra-operator reliability results were observed when the examined muscle was on the patient’s side opposite to the examiner. This again may be the result of the different pressures applied by the probe and thus again raises the question on methodology [29].

## 4. Elastography in Different Muscle Disorders

The following subparagraphs focus on the elastography results in relation to the patient’s muscles disease. The muscular dystrophies, other myopathies, cerebral palsy and its treatment are discussed in turns.

### 4.1. Muscle Elastography in Muscular Dystrophies

As research on treatment strategies for myopathies has been progressing over the recent years, the need for treatment monitoring tools is also increasing. Few studies have presented differences between healthy peers and patients with DMD. Lacourpaille, Lilian, et al. compared results from different muscles in a healthy group (*n* = 13) and a DMD group (*n* = 14) of patients aged 5–24 years. While significant differences in muscle stiffness were found in the AT, GCM, vastus lateralis, biceps, and triceps brachii, the difference in muscle stiffness of the abductor digiti brevis minimi did not reach statistical significance. The highest difference was observed in the vastus lateralis, with the stiffness 134% higher in the DMD group [21]. Pichiecchio et al. compared the SWE and MRI results of the lower limb muscles (GCM, AT, rectus femoris, vastus lateralis and medialis, adductor magnus, and gluteus maximus) from 5 children with DMD with those from their age-matched healthy peers. Moderately higher muscle stiffness values were found in the DMD group. However, no significant correlation was found between the SWE and MRI results for fat replacement and muscle oedema on T1 and short inversion-time inversion-recovery (STIR) images. Although the study was limited by both the small number of participants and inclusion of a patient with a milder clinical presentation and mutation associated with Becker dystrophy, it presented the question of whether MRI and SWE analyse overlapping or different aspects of muscle diseases and if they can be used interchangeably. In addition, the patient with a clinical presentation of Becker dystrophy showed interesting results in that the changes in the SWE values were not accompanied by fat or STIR changes on MRI and clinical abnormalities in the patient examinations, which possibly preceded the latter [4]. The hypothesis that elastography might be useful as a screening tool thus remains to be proven.

Furthermore, by analysing changes in SWE results from patients with DMD over 12 months, Lacourpaille proved that elastography is a good candidate monitoring tool. They compared resting shear modules from the AT, GCM, biceps and triceps brachii, and abductor digiti minimi muscles in 10 children with genetically confirmed DMD and 9 age-matched healthy peers. While no significant changes over time were found in the control group, the DMD group showed significant increases in the AT (75.1% ± 93.5%, *p* = 0.043), GCM (144.8% ± 180.6%, *p* = 0.050), and triceps brachii (35.5% ± 32.2%, *p* = 0.005). The biceps brachii and abductor digiti minimi muscle changes did not reach statistical significance [32].

### 4.2. Other Myopathies

Berko et al. designed a study to evaluate the usefulness and efficacy of SE for juvenile idiopathic inflammatory myopathies (JIIMs) and compared them with those of MRI. The authors recruited 18 patients aged 3 to 19 years, assessed the clinical activity of the disease, and performed both MRI of the pelvic region and thighs and strain elastography of the quadriceps muscles. The results were not favourable for SE. While the MRI results were related to disease activity (*p* = 0.012), the elastography results showed no association with either the MRI results or disease activity. Also no relationship was found between elastography and disease duration; thus, the results cannot be simply explained by the shorter disease time in the adults [5]. The question remains as to whether qualitative and less operator-dependent elastographic modalities would provide better results, as JIIMs are expected to affect muscle elasticity.

By contrast, Song et al. examined the SEs of patients with inflammatory myopathies, regardless of age. They proved that the affected muscles had higher strain rates and that SR correlated with the pathological scores of the biopsy samples [12]. However, as only one of the 17 patients was a child (an 11-year-old girl with juvenile dermatomyositis) and her SR was lower than those of the other participants, no conclusion for the paediatric population may be given from this study. This also implies the possibility that age influences elastography results.

### 4.3. Cerebral Palsy

CP is one of the most common disorders associated with secondary muscle changes in the paediatric population. Owing to the large number of patients, the patterns of changes found in affected muscles, and the treatments aimed at decreasing muscle spasticity, only a few studies on elastography in CP already exist.

Few studies focused on assessing the differences between muscles affected and those not affected by CP. Kwon at al compared strain and ARFI results from the GCM and soleus muscle in 15 patients with CP and 13 healthy peers, all aged <13 years. The GCM had greater muscle stiffness on SE and higher ARFI velocity in the CP group. While the soleus muscle had higher values on SE, its shear wave velocity was similar in both the CP and healthy groups. The SR (ratio of the GCM to the soleus muscle) was significantly higher in the CP group than in the healthy group. The authors concluded that the GCM had greater involvement in the motor deficits in CP [33]. In another study, Lee at al., based on a group of 7 children with hemiplegic CP and 1 post-stroke paediatric patient with similar clinical presentations, proved that the AT and GCM had significantly different SW speeds between the less and more affected sides. Patients with Gross Motor Function Classification System levels I and II, indicating no gross motor deficits, were compared. In the neutral joint position, the mean SW speed was 20% higher for the AT on the more affected side (3.86 m/s vs. 3.22 m/s, *p* = 0.03) and 14% higher for the GCM (5.04 m/s vs. 4/46 m/s, *p* = 0.024) [26]. Ozturk et al. compared Achilles tendon stiffness between CP patients (72 children, CP group) and their healthy peers (83 children, control group). The control group had lower SR than the CP group (1.7 ± 0.1 vs. 4.1 ± 0.8, *p* < 0.001) [22]. On the basis of the SWEs of the soleus muscle in 21 children with CP and 21 healthy peers, Vola et al. proved that muscle elasticity differed significantly between the hemiplegic and healthy patients. Higher Young modulus values were found in the CP group than in the healthy group (8.1 ± 2.3 kPa vs. 4.8 ± 1.7 kPa, *p* < 0.001) [34]. Similar results were presented for ARFI by Bilgici et al. In their study in 17 children with CP and 25 healthy peers, they compared GCM elasticity and modified Ashworth scale (MAS) scores. The mean shear wave velocity was 3.17 ± 0.81 m/s in the CP group and 1.45 ± 0.25 m/s in the control group (*p* < 0.001) [35]. A recent study by Lallemant-Dudek et al. compared SWE results of GCM and biceps brachii long. Paper has some important limitations as the control group included patients with scoliosis while no information on possible muscle disease was given and in addition some of the patients from CP did receive botulin toxin (BoNT-A) treatment. Yet authors found that while there was no difference between groups when muscle was at rest, CP involved muscles differed from both–control group and uninvolved muscles in CP patients [36]. In other study, Mansouri et al. confirmed the relationship of the elastography results of the anterior tibialis muscle and GCM with gait abnormalities (step time and walking speed) [28]. Some studies confirmed that muscle stiffness assessed using elastography is related with clinical presentations assessed using MAS score [34,35]. While in others no such correlation was found [36].

To test the hypothesis that hyperactivity and spasticity of the hip abductors and flexors result in the hip displacement in CP, Doruk Analan et al. analysed the correlation between the Reimers hip migration index and the elasticity of the mentioned muscles on SWE. No significant correlation was found [37].

### 4.4. CP Treatment Evaluation Using Elastography

The use of elastography in monitoring botulin toxin A (BoNT-A) treatment in patients with CP is gaining attention. Studies that showed changes in muscle stiffness after BoNT-A administration into GCMs in children are presented in Table 1. In addition, some authors presented clear associations between post-BoNT-A changes in elastography result and clinical scale scores for spasticity (MAS and modified Tardieu scale [MTS]) [27,35,38]. By assessing post-BoNT-A elasticity changes of the anterior tibialis in addition to those of the gastrocnemius, Dag et al. proved that botulin treatment affects not only the muscle where BoNT-A was administrated but also the overall patient gait and related muscles. In some patients, paradoxical increases in shear wave speed were observed regardless of changes in MAS score, probably due to abnormal collagen content, injection failure, wrong injection site, insufficient dose, and so forth. In such cases, measurement of changes between the pre- and post-elastography values combined with US might prove to be a useful tool for further decision making regarding eventual treatment withdrawal [39]. In addition to mentioned studies that focused on the treatment effects at one month post BoNT-A administration, in a study in 9 children aged 2 to 9 years, Brandenburg et al. quantified the duration of treatment effect. They set three study visits up to 1 month before and 1 and 3 months after injection. While near-significant differences were found between the pre-BoNT-A administration and first post-treatment control, no significant difference was found between the pre- and 3-month post-BoNT-A values. The most significant difference was found between the 1- and 3-month post-BoNT-A values. Thus, the authors showed that the BoNT-A effect on muscle stiffness on SWE lasted <3 months post injection. SWE proved to be a reliable tool for individualised monitoring and planning of botulin treatment in patients with CP [40]. We can then conclude that elastography might be useful in the most effective and patient-tailored BoNT-A therapy by guiding administration planning, determining the treatment prognosis, allowing for objective treatment assessment (e.g., comparison between elastography-based MAS and MTS scores less subjectively and isolated muscle assessment rather than combined assessment of muscles, joints, tendons, surrounding tissues, and excitability changes), and guiding the choice of the best time interval between doses.

## 5. Muscle Elastography in Other Diseases

The following subparagraphs focus on the elastography results in relation to other diseases influencing muscle tissue. The chronic kidney diseases, gluteus muscle contracture, torticollis, Osgood-Schlatter disease, elbow injuries, musculoskeletal tumours are be discussed in turns.

### 5.1. Chronic Kidney Diseases

In their study, Bekci et al. assessed the possible use of ARFI in screening for muscle changes in chronic kidney diseases (CKDs). The reason for the muscle function loss in CKD is still not fully elucidated, but factors such as disease-related myopathy, muscle loss, and abnormal fat deposition are considered possible causes. The study population consisted of children aged 6 to 17 years, including 23 patients with CKD (11 girls) and 22 healthy peers (11 girls). The authors performed an elastographic evaluation of the elasticity of the rectus femoris muscle and handheld dynamometry (HHD) for evaluation of the maximal isometric strength of the knee extensors. The results showed that both muscle strength and elasticity were significantly decreased in the CKD group compared with the healthy volunteers. Whereas HHD has limited reliability, the authors concluded that elastographic techniques might prove feasible, affordable, and objective tools for treatment planning, monitoring, and screening for muscle changes [35].

### 5.2. Gluteus Muscle Contracture

Gluteus muscle contracture is a clinical syndrome most often found in the age group of 6–18 years, in relation to the above-mentioned pathological muscle changes, and is characterised by abnormal gait and hip movement limitations (mainly flexion and adduction). Diagnosis is often delayed and thus affects the prognosis, which is closely related to early treatment (surgery being the gold standard). In a small group of three patients, Guo et al. proved that measurement of muscle stiffness using SWE might be useful in the diagnosis. They speculated that SWE results may be related to the severity of the syndrome, making elastography a potentially useful tool for the assessment needed for treatment and prognosis [41].

### 5.3. Torticollis

Lee at al performed strain elastography of the sternocleidomastoid muscle and compared the results between infants with congenital torticollis and their healthy peers. The torticollis group had significantly lower muscle elasticity values [31]. The authors concluded that elastography may be a useful tool for monitoring and diagnosing torticollis especially in cases with subtle changes.

### 5.4. Osgood-Schlatter Disease

On the basis of real-time tissue elastography results of the rectus femoris muscles in 37 teenage male athletes, Enomoto et al. rejected the hypothesis that one of the factors that lead to Osgood-Schlatter disease is higher-than-normal muscle stiffness. No significant difference in quadriceps muscle stiffness was found between the OGD and healthy groups [2].

### 5.5. Elbow Injuries Related to Sports

In a study based on strain elastography results of the upper limb muscles in 197 baseball players aged 9 to 15 years, Saito et al. focused on addressing the question as to whether elbow injuries are related to pronator teres muscle (PTM) stiffness. The muscle group functions as a dynamic stabiliser against elbow valgus force. According to US results, the participants were divided into three groups, namely those with medial epicondylar fragmentation in the throwing arm, those with osteochondritis dissecans (OCD) of the humoral capitellum, and healthy peers. The elasticity of the pronator teres muscle was significantly higher in both injury groups than in the healthy group. In addition, the authors found that while only the muscle spasticity of the throwing arm was significantly higher in the OCD group, the PTMs on both sides were affected in the medial elbow injury group. The authors concluded that this may prove that medial elbow injury might be the result of muscle changes, not the other way around; thus, screening for muscle stiffness changes might help prevent the injury. Moreover, by comparing the strain results between the range of movement of the upper limb and those of the elbow and arm joints (with significant differences only for external rotation of the glenohumeral joint), the authors remarked that the changes in muscle spasticity observed on elastography might precede those observed in clinical examinations [30].

### 5.6. Oncology: Musculoskeletal Tumours

Timely detection and diagnosis of suspicious lesions are often the factors that lead to better treatment efficacy. However, the heterogeneity of pathological masses challenges all available techniques. Li et al. evaluated the usefulness of real-time 2-D SWE in distinguishing between benign and malignant musculoskeletal lesions by examining 115 tumours in 92 children and adults. Both quantitative (minimum, maximum, and mean elasticity in kilopascals) and qualitative (colour map sale) elastography results were analysed in comparison with the histopathological evaluation results. All the parameters were significantly different between the benign and malignant tumours (*p* < 0.05). By performing a multivariate regression analysis, the mean elasticity values were found to have strongest independent prediction for malignancy, with 71.4% accuracy, 66.7% sensitivity, and 85% specificity. In the same study, the authors also compared the diagnostic efficacy of US with those of both qualitative and quantitative SWEs. They found no significant differences in diagnostic efficacy, which was considered moderate for all the techniques. Assessment of lesion stiffness proved to be an important addition in morphological evaluations. One of the important limitation of this study was the exclusion of tumours exceeding the maximal region of interest of 4 × 6.5 cm [42].

## 6. Study Limitations and Conclusions

Since most existing studies have different study protocols in relation to the different positions of the analysed limb/muscles, which affected the acquired measurements; the stretching protocol; the non-homogenous groups with regard to age and sex; and the lack of standardisation of the pressure generated by the transducers on the patients’ skin and muscles, the results of the studies included in our analysis were not comparable in most aspects.

Further studies are needed to develop normative values for different age groups that account for developmental changes, to characterise the influences of sex on the normative values, to standardise the test protocol, and to assess whether skin and tissue compression significantly changes measured values. Moreover, further investigation into the probe position on muscles (perpendicular and distal/proximal positions), the influence of body temperature, muscle stretching, different muscles, and the relationships of stiffness values to clinical characteristics (e.g., hypotonia) and other diagnostic tools are needed. Nevertheless, application of this imaging modality is a promising direction for the diagnosis and monitoring of muscular diseases. Development of unified examination protocols and further objectification of muscle elastography may be an important step for better understanding, recognition, and monitoring of the different muscle pathologies in specific, non-easily cooperating group of small patients.

## Figures and Tables

**Table 1:** Studies on the relationship between elastography and BoNT-A treatment in cerebral palsy.

Study	Method	Population	Muscle Assessed	Before BoNT-A		1 Month after BoNT-A	*p* Value
Ceyhan Bilgici et al., 2018 [39]	ARFI	*n* = 12 (6♀) 8.58 ± 2.48 yo	Gastrocnemius	SWS: 3.20 ± 0.14 m/s		SWS: 2.45 ± 0.21 m/s	<0.01
Park and Kwon, 2012 [38]	SE	*n* = 17 (7♀) 4.75 ± 1.83 yo	Medial gastrocnemius	RTS score: 3.4		RTS score: 1.5	<0.05
Dağ et al., 2020 [27]	SWE	*n* = 24 (10♀) 2–11 yo	Lateral gastrocnemius	Stiffness: 45.9 ± 6.5 kPa		Stiffness: 25.0 ± 5.7 kPa	<0.01
			Anterior tibialis	Stiffness: 36.9 ± 7.9 kPa		Stiffness: 28.4 ± 5.2 kPa	<0.01
Brandenburg et al., 2018 [40]	SWE	*n* = 9 (4♀) 2–9 yo	Lateral gastrocnemius	0° PF	1 month vs. 3 months after BoNT-A*		0.02
				10° PF			0.03

ARFI, acoustic radiation force impulse; BoNT-A*, botulin toxin A; PF, plantar flexion; RTS, real-time sonoelastography; SE, strain elastography; SWE, shear wave elastography; SWS, shear wave speed; yo, years old.
